# Ultrathin ALD Metal Oxide Coatings Improve the Triboelectric Performance of Regenerated Cellulose

**DOI:** 10.3390/nano16130786

**Published:** 2026-06-23

**Authors:** Christina Dahlström, Erfan Jafarpour, Alireza Eivazi, Renyun Zhang, Jesper Edberg, Ioannis Petsagkourakis, Laura Keskiväli, Jukka A. Ketoja, Magnus Norgren

**Affiliations:** 1 Surface and Colloid Engineering, FSCN Research Centre, Mid Sweden University, Holmgatan 10, 85170 Sundsvall, Swedenalireza.eivazi@miun.se (A.E.); jukka.ketoja@vtt.fi (J.A.K.); magnus.norgren@miun.se (M.N.); 2 Material Physics, FSCN Research Centre, Mid Sweden University, Holmgatan 10, 85170 Sundsvall, Sweden; renyun.zhang@miun.se; 3 Smart Hardware, Bio and Organic Electronics, RISE Research Institutes of Sweden, Södra Grytsgatan 4, 60233 Norrköping, Sweden; jesper.edberg@ri.se (J.E.); ioannis.petsagkourakis@ri.se (I.P.); 4 Digital Cellulose Center, 60233 Norrköping, Sweden; 5 VTT Technical Research Centre of Finland Ltd., Tietotie 21, 02150 Espoo, Finland; laura.keskivali@vaisala.com

**Keywords:** regenerated cellulose, triboelectric nanogenerators, atomic layer deposition

## Abstract

Regenerated cellulose is a promising tribopositive material for sustainable triboelectric nanogenerators (TENGs), although its electrical output remains sensitive to surface and interfacial properties. In this study, regenerated cellulose was modified using atomic layer deposition (ALD) of Al_2_O_3_, TiO_2_, and ZnO to investigate how nanoscale oxide coatings influence triboelectric performance against a tribonegative PTFE counter layer. Two deposition regimes were examined: 7 ALD cycles, representing the early stage of ALD growth, and 200 cycles, representing a more developed coating regime. Triboelectric measurements, dielectric spectroscopy, structural characterization and contact angle analysis, were used to evaluate how ALD modification influences the electrical response of regenerated cellulose. All ALD-modified samples exhibited increased surface charge density and power output compared to unmodified cellulose, while also showing improved retention of triboelectric performance at elevated relative humidity. The 7-cycle samples consistently outperformed the corresponding 200-cycle coatings under low-humidity conditions, whereas the 200-cycle ZnO sample exhibited the highest humidity stability. No direct correlation between wettability and triboelectric output was observed. The results suggest that relatively small interfacial modifications introduced by ALD are sufficient to influence both the triboelectric response and humidity-dependent charge dissipation behavior of regenerated cellulose.

## 1. Introduction

Triboelectric nanogenerators (TENGs) have emerged as a promising technology for harvesting mechanical energy for self-powered sensors and sustainable energy systems, due to their simple architecture, material versatility, and scalability [1]. A TENG converts mechanical motion into electrical energy through contact electrification and electrostatic induction [2]. Increasing interest in sustainable and biodegradable materials has driven the exploration of cellulose as an alternative to conventional petrochemical-based polymers [3,4,5,6]. Regenerated cellulose, in particular, combines renewability, mechanical flexibility, and a favorable position in the triboelectric series, making it an attractive candidate for more sustainable TENG applications [7,8,9].

Despite these advantages, the triboelectric performance of cellulose remains sensitive to ambient conditions, particularly humidity-induced charge dissipation and limited charge retention, which reduce both the electrical output and environmental stability of cellulose-based TENG systems [10,11,12]. At the same time, strategies for improving the electrical output of cellulose-based TENGs remain of considerable interest. These challenges highlight the importance of controlling surface and interfacial properties, which govern charge generation and stability in triboelectric systems.

Atomic layer deposition (ALD) provides a versatile approach for modifying surface and interfacial properties through conformal coatings with sub-nanometer thickness control and well-defined surface reactions [13]. The self-limiting growth mechanism of ALD allows uniform coatings on complex and porous substrates, which makes it particularly suitable for cellulose-based materials. In triboelectric systems, ALD has mainly been explored in specific cases, e.g., using TiO_x_ coatings on polytetrafluoroethylene (PTFE) to improve environmental stability and reduce humidity-induced charge dissipation [14]. More recently, ALD and vapor-phase infiltration approaches have also been applied to porous polyvinylidene fluoride (PVDF)-based TENG systems to tailor interfacial charge transport and humidity-responsive triboelectric behaviour [15]. Ultrathin metal oxide coatings introduced by ALD can also influence dielectric properties and interfacial polarization, which in turn affect charge generation and electrostatic coupling in TENGs [14,15].

For cellulose-based materials, ALD has been used to deposit conductive oxide layers such as aluminum-doped ZnO on cellulose nanofibril papers for flexible electronics and TENG devices [16]. Previous studies have shown that the first few ALD cycles can significantly alter the wettability of cellulose surfaces. For example, Al_2_O_3_ and ZnO coatings on cellulose fibers initially lead to a more hydrophobic surface, while increasing the number of cycles can restore hydrophilic behavior [17]. A similar trend was observed for TiO_2_, where a small number of ALD cycles changes a hydrophilic surface to hydrophobic [18]. However, even though these studies show clear changes in surface wettability, it is still unclear how this affects triboelectric performance. The relationship between surface modification, dielectric behavior, and triboelectric output has not been systematically investigated for cellulose-based systems.

In this work, regenerated cellulose films were modified using ALD-deposited Al_2_O_3_, ZnO, and TiO_2_ at two deposition regimes: 7 cycles and 200 cycles. Low cycle numbers have been associated with more hydrophobic surface behavior, while increasing the number of cycles beyond approximately 20 cycles lead to a transition back toward more hydrophilic behavior [17,18]. Therefore, 200 cycles were selected to represent a substantially more developed coating regime well beyond this reported transition region. The ALD-modified cellulose films were evaluated as the tribopositive layer against tribonegative PTFE in a contact–separation TENG configuration.

We hypothesize that the number of ALD cycles influences the triboelectric response by changing the surface and near-surface properties of regenerated cellulose. Low cycle numbers may be sufficient to affect surface charging, while higher cycle numbers may introduce changes in the dielectric environment that influence the output.

By combining triboelectric measurements with dielectric spectroscopy and surface characterization, this study aims to assess how differences in ALD cycle number influence performance. The results show that the investigated ALD regimes influence both the triboelectric performance and humidity-dependent stability of regenerated cellulose-based TENGs.

## 2. Materials and Methods

### 2.1. Raw Materials and Chemicals

Dissolving pulp was supplied by Domsjö Fabriker Aditya Birla (Örnsköldsvik, Sweden). The weight-average molecular weight (M_w_) was 3.2 × 10^5^ g mol^−1^, the limiting viscosity was 432 mL g^−1^, the total hemicellulose content was 4.5 wt%, and the polydispersity index was 10.3, as determined by size exclusion chromatography.

The polytetrafluoroethylene (PTFE) film (130 μm thick) was purchased from High-tech-flon^®^ (Konstanz, Germany), and the copper tape was delivered by Elektrokit (Malmö, Sweden). Urea (≥99.5%) was purchased from Sigma-Aldrich (Solna, Sweden), ethanol (≥99.8%) from VWR Chemicals (Stockholm, Sweden), and LiOH·H_2_O (56.5%) from Alfa Aesar (Karlsruhe, Germany).

### 2.2. Cellulose Dissolution and Film Preparation

The cellulose fibers were dissolved in an aqueous solution containing 4.6 wt% LiOH and 15 wt% urea. The solution was first frozen and thawed to −12 °C, after which 4.0 wt% dissolving pulp was added and mixed using a mechanical stirrer at 1300 rpm for 2 min. The freezing, thawing, and mixing procedure was repeated twice to ensure complete dissolution.

The cellulose solution was subsequently centrifuged at 8000 rpm for 10 min to remove undissolved fibers, air bubbles, and possible contaminants. The solution was cast onto a glass plate and immersed in an ethanol regeneration bath for 2 h. The regenerated films were then immersed in Milli-Q water for three days with daily water exchange. Finally, the films were dried using a Rapid-Köthen paper-making system (Frank-PTI GmbH, Birkenau, Germany) at 90 °C and −0.95 bar for 10 min.

The regenerated cellulose films are referred to as R-cellulose throughout this work.

### 2.3. Atomic Layer Deposition (ALD)

Al_2_O_3_ and ZnO ALD modifications were performed using a Picosun Sunale Standard R-200 reactor (Picosun Oy, Espoo, Finland) in single-wafer mode. N_2_ (>99.999%) was used as carrier and purge gas. Al_2_O_3_ was deposited using trimethylaluminium (TMA, >99%, Strem Chemicals, Newburyport, MA, USA) and deionized water as precursors with pulse/purge times of 0.2/11/0.2/11 s (TMA/purge/H_2_O/purge). ZnO was deposited using diethyl zinc (DEZ, >95%, Strem Chemicals, Newburyport, MA, USA) and deionized water with pulse/purge times of 0.3/11/0.2/11 s (DEZ/purge/H_2_O/purge). The deposition temperature for Al_2_O_3_ and ZnO was 150 °C.

TiO_2_ ALD modifications were produced using a Picosun Sunale R-200 Advanced reactor (Picosun Oy, Espoo, Finland). N_2_ (>99.999%) was used as carrier and purge gas. TiO_2_ was deposited using titanium tetrachloride (TiCl_4_, 99%, Strem Chemicals, Newburyport, MA, USA) and deionized water as precursors at a deposition temperature of 120 °C.

**Figure 1:**
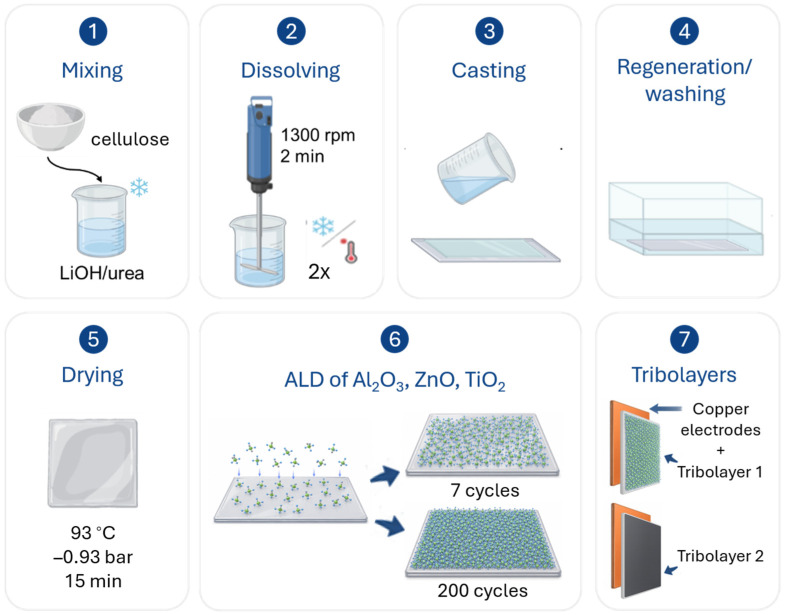
Schematic illustration of the regenerated cellulose film preparation, atomic layer deposition (ALD) surface modification, and tribolayer assembly process. The figure was created with BioRender.com.

For all oxides, the number of ALD cycles varied between 7 and 200 cycles. The sample names for the ALD-modified cellulose films are based on the number of cycles and deposited oxide, e.g., 7 ZnO and 200 ZnO. A schematic illustration of the sample preparation is shown in Figure 1.

The sample thickness of the R-cellulose and ALD-modified cellulose films was measured at ten different positions using a micrometer screw gauge, and the reported values represent the mean thickness (Table 1).

### 2.4. Structural and Surface Characterization

Scanning electron microscopy (SEM) imaging of the R-cellulose and ALD-modified samples was performed in secondary electron mode using a TESCAN MAIA3 electron microscope (TESCAN Group a.s., Brno, Czech Republic). Prior to imaging, the samples were coated with a 2 nm iridium layer using a Quorum Q150T ES sputter coater (Quorum Technologies Ltd., Laughton, East Sussex, UK).

X-ray diffraction (XRD) measurements were performed at room temperature using a Bruker D2 Phaser diffractometer (Bruker, Billerica, MA, USA) equipped with Cu Kα radiation (*λ* = 1.54 Å), operating at 30 kV and 10 mA in θ–2θ configuration. The step size was 0.04°.

The film samples were mounted on specially cut silicon single-crystal substrates to minimize background scattering and avoid interfering diffraction peaks.

Atomic force microscopy (AFM) was used to evaluate the surface roughness of the R-cellulose and ALD-modified samples. Measurements were carried out using a Park Systems NX20 instrument (Park Systems Corp., Suwon, Republic of Korea) operating in non-contact mode. A PPP-NCHR probe (Park Systems Corp., Suwon, Republic of Korea) with a nominal resonance frequency of 330 kHz and a force constant of 42 N m^−1^ was used.

AFM images were acquired over representative areas of 10 × 10 μm^2^ at scan rates between 0.7 and 0.9 Hz, ensuring more than 95% agreement between forward and backward scans. The surface roughness parameter (*R_q_*) was calculated using Park Systems XEI software (version 1.8.5).

Fourier-Transform Infrared Spectroscopy (FTIR) spectra were collected using a Thermo Scientific Nicolet 6700 spectrometer (Thermo Fisher Scientific Inc., Madison, WI, USA) equipped with a Smart Orbit diamond attenuated total reflectance (ATR) accessory. Spectra were recorded in the range of 400–4000 cm^−1^ with a resolution of 2 cm^−1^ and averaged over 32 scans.

Static water contact angle measurements were performed using a Theta Lite Optical Tensiometer (Biolin Scientific AB, Västra Frölunda, Sweden) at room temperature. A 4 µL droplet of deionized water was deposited onto the sample surface using a microsyringe.

The contact angle was monitored between 0 and 10 s after droplet deposition. The reported values correspond to measurements obtained 1 s after droplet deposition to minimize the influence of initial droplet oscillation and spreading while avoiding effects associated with liquid absorption at longer times. Contact angles were determined using the Young–Laplace fitting method provided by the instrument software.

### 2.5. Dielectric Characterisation

Dielectric spectroscopy measurements were performed using an Alpha High Resolution Dielectric Analyzer (Novocontrol Technologies GmbH & Co. KG, Montabaur, Germany) in the frequency range of 1 Hz–10 MHz. Gold electrodes were evaporated onto both sides of the samples using shadow masks with a diameter of 10 mm. The samples were cut into circular discs with a diameter of 16 mm.

Prior to measurement, the samples were conditioned at 50% RH in a climate chamber for 24 h and subsequently encapsulated into coin cells. The edges of the coin cell spacer were coated with a dielectric material to define the active electrode area (10 mm diameter) and prevent short circuits.

### 2.6. Triboelectric Measurements

Triboelectric measurements were performed by attaching the sample to copper tape mounted on a linear motor. The counter tribolayer consisted of a PTFE film attached to copper tape and mounted on the opposing side of the linear motor. The contact–separation speed was set to 0.3 m s^−1^ and the operating frequency to 4.5 Hz. The active contact area of the TENG setup was 3 × 3 cm^2^.

Electrical signals were recorded using a Keysight DSOX3024T oscilloscope equipped with a 100 MΩ probe (Keysight Technologies, Santa Rosa, CA, USA).

The output power of the TENG was evaluated using two different approaches. In the first approach, a simplified estimate of the peak electrical output was obtained by multiplying the maximum open-circuit voltage (*V*_max_) and maximum short-circuit current (*I*_max_) obtained during contact–separation operation. This value was used for comparative purposes between samples. In the second approach, the load-dependent power output was determined by measuring the voltage and current across external load resistors. The power (*P*) was calculated according to:

(1) $$P=RI^{2},$$

where *R* is the external load resistance and *I* is the measured current.

The temperature during the measurements was 21.1 ± 0.2 °C and the relative humidity (RH) was 30.6 ± 1.0% RH.

The surface charge density (*σ*) was calculated from the short-circuit current data obtained during TENG measurements by integrating the current peak corresponding to the pressing cycle between times *t*_1_ and *t*_2_ and dividing by the contact area (*A*) [19]:

(2) $$\sigma =\frac{Q}{A}=\frac{\int_{t_{1}}^{t_{2}}I(t)dt}{A},$$

where *Q* is the maximum transferred charge.

For each short-circuit current dataset, eight individual current peaks were integrated to calculate the mean surface charge density. For every sample type (e.g., 7 ZnO and 200 ZnO), two independent samples were measured, and the reported values represent the average of these measurements.

For the investigation of the effect of relative humidity on the triboelectric performance of the samples, the relative humidity was adjusted to determined values between 10% and 90% RH (at 10% intervals) using a Cellkraft P10 humidifier (Cellkraft AB, Stockholm, Sweden) and each sample was analyzed.

## 3. Results and Discussions

This study investigates how ALD-deposited metal oxide coatings on regenerated cellulose influence triboelectric performance. To capture differences between early and more developed stages of ALD growth, samples prepared with 7 and 200 cycles were compared. Initial ALD growth is often characterized by discrete nucleation sites and island growth until coalescence occurs at higher cycle numbers [20], and therefore both 7 and 200 cycles are included in this study. The results are presented by first examining surface and structural properties, followed by dielectric behavior and triboelectric performance.

### 3.1. Surface and Structural Properties

**Figure 2:**
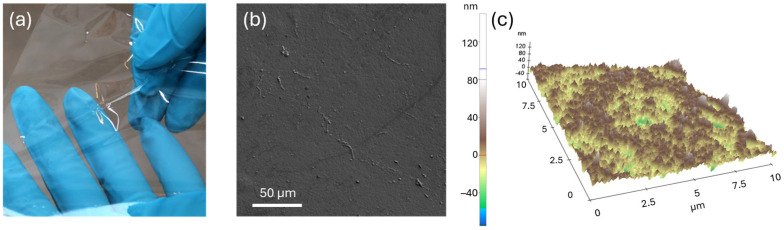
(**a**) A photograph showing the R-cellulose film and corresponding (**b**) scanning electron microscopy (SEM) image of the surface and (**c**) atomic force microscopy (AFM) 3D height image.

The regenerated cellulose (R-cellulose) films are fully transparent (Figure 2a), and this is maintained after ALD treatment. Surface morphology was characterized at multiple length scales. SEM images were used to assess macroscopic surface uniformity, which can influence the effective contact area during TENG operation, while AFM measurements (10 × 10 µm^2^) provided information on nanoscale surface topography.

As shown in Figure 2b, the R-cellulose surface appears relatively smooth at the micrometer scale. The AFM image (Figure 2c) further shows that the surface exhibits low nanoscale roughness, with an average roughness (*R_q_*) of 14.7 nm.

**Figure 3:**
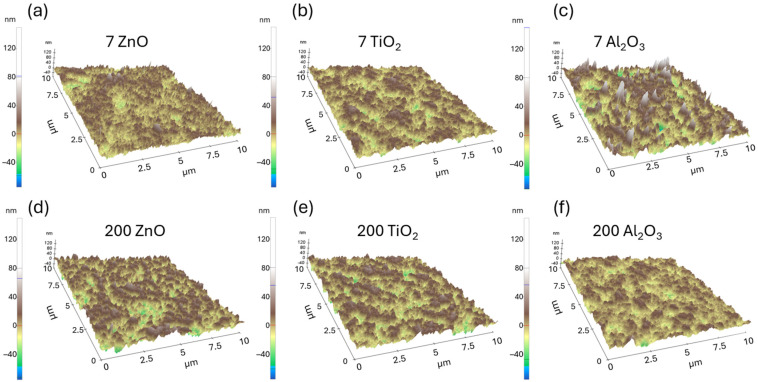
AFM 3D-height images of the ALD samples; (**a**) 7 ZnO, (**b**) 7 TiO_2_, (**c**) 7 Al_2_O_3_, (**d**) 200 ZnO, (**e**) 200 TiO_2_ and (**f**) 200 Al_2_O_3_.

After ALD, the surface roughness varies slightly depending on the deposited material and number of cycles (Table 1 and Figure 3). However, the changes are relatively small. The R-cellulose exhibits an average roughness of ~15 nm, while the ALD-treated samples fall within ±~4 nm of this value, without a clear trend.

**Figure 4:**
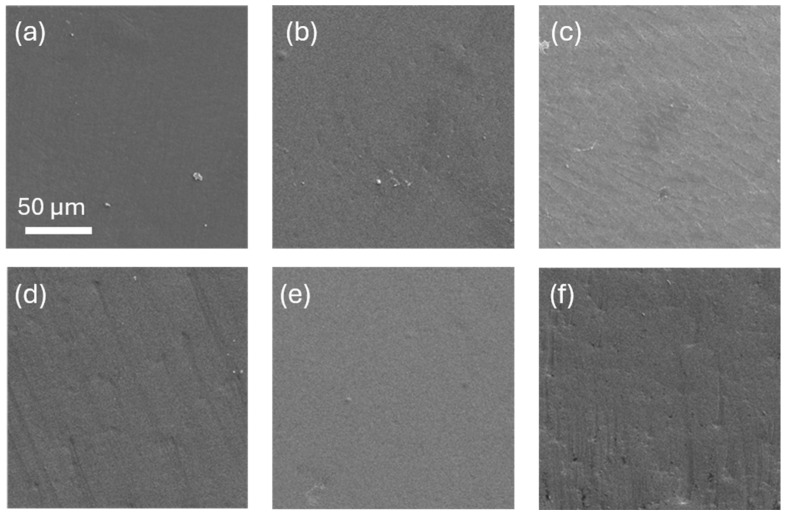
SEM images of the ALD-modified cellulose films: (**a**) 7 Al_2_O_3_, (**b**) 7 TiO_2_, (**c**) 7 ZnO, (**d**) 200 Al_2_O_3_, (**e**) 200 TiO_2_, and (**f**) 200 ZnO.

The SEM images (Figure 4) similarly show no noticeable changes in the overall surface morphology after coating. These results suggest that the ALD process does not significantly alter the overall surface profile at these length scales.

In contrast to the surface morphology, thickness measurements show a clear increase after ALD treatment (Table 1). The measured thickness includes both the cellulose substrate and the contribution from the ALD treatment and therefore does not directly represent the actual oxide coating thickness alone. The observed increase in total film thickness (16–39%) is substantially larger than expected from nominal ALD growth, which is typically reported to be approximately 0.1–0.2 nm per cycle for these oxide systems depending on deposition conditions and precursor chemistry [21,22,23]. This translates into a thickness of ~0.7–1.4 nm for 7 cycle deposition and ~20–40 nm for 200 cycle deposition.

The observed thickness increase is consistent with partial precursor infiltration and/or swelling of the cellulose structure during the ALD process, phenomena previously reported for ALD on polymeric and cellulose-based fibres [24]. The magnitude of this increase is comparable to that observed in cellulose films exposed to high relative humidity (>70%) [25], suggesting that similar interactions may occur between the cellulose matrix and ALD precursors.

**Figure 5:**
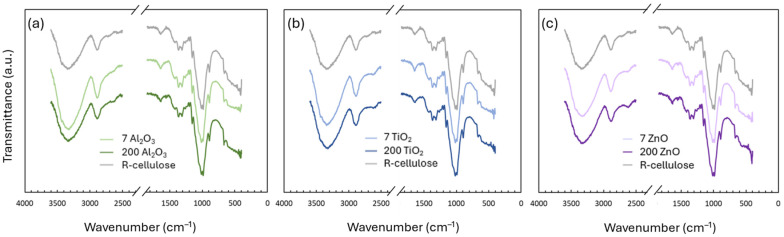
Fourier-Transform Infrared Spectroscopy (FTIR) spectra of the R-cellulose and the different ALD samples, (**a**) Al_2_O_3_, (**b**) TiO_2_ and (**c**) ZnO.

The FTIR spectra (Figure 5) of the TiO_2_-, Al_2_O_3_-, and ZnO-coated samples retained the characteristic absorption bands of cellulose II, including the broad O–H stretching region (~3100–3500 cm^−1^), C–H stretching vibrations (~2980–2835 cm^−1^), and bands associated with C–O and C–O–C vibrations in the fingerprint region [26]. The overall spectral features remain similar to those of the reference cellulose sample, indicating that the cellulose backbone structure is preserved after ALD processing.

However, the ALD-modified samples exhibit stronger absorption in the broad O–H stretching region (~3300 cm^−1^) compared to the uncoated R-cellulose, indicating an increased contribution from hydroxyl- and hydrogen-bonded species within the IR sampling depth, such as metal–OH groups and/or associated water. The strongest absorption in the O–H stretching region is observed for the 7-cycle Al_2_O_3_ sample, followed by 7 TiO_2_ and 7 ZnO, which follows the same trend as the observed thickness increase (Al_2_O_3_ > TiO_2_ > ZnO).

**Figure 6:**
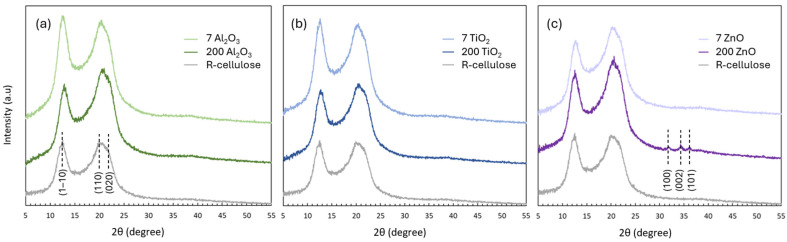
X-ray diffraction (XRD) patterns of the R-cellulose and the different ALD samples, (**a**) Al_2_O_3_, (**b**) TiO_2_ and (**c**) ZnO.

The XRD patterns (Figure 6) show that the characteristic cellulose II peaks are retained after ALD treatment, indicating that the crystalline structure of the cellulose is preserved.

For Al_2_O_3_ and TiO_2_ (Figure 6a,b), no additional diffraction peaks are observed, even for the 200-cycle samples, indicating that no crystalline anatase or rutile TiO_2_, nor γ- or α-Al_2_O_3_ phases, are formed under the applied conditions [27]. At the relatively low ALD temperatures used in this study, Al_2_O_3_ and TiO_2_ are expected to remain predominantly amorphous. Crystallization of Al_2_O_3_ typically requires substantially higher temperatures, and TiO_2_ may crystallize into anatase at lower temperatures, commonly above ~150–250 °C depending on precursor chemistry, deposition temperature, film thickness, impurities, and post-deposition annealing conditions [28]. Therefore, the absence of crystalline Al_2_O_3_ and TiO_2_ peaks in XRD is consistent with low-temperature ALD oxide growth.

For ZnO, the 7-cycle sample similarly shows no detectable ZnO-related reflections. In contrast, weak peaks appear in the 200-cycle sample in the 2θ ≈ 31–37° range, consistent with the reflections of wurtzite ZnO. This is in line with previous reports showing that a certain number of ALD cycles is required before crystalline domains become detectable [29]. Perrotta et al. (2020) reported that 20 cycles are required for crystallites to form which agrees with the results where the 200 cycles show peaks and the 7 cycles do not [29].

The contact angle measurements (Table 1) show that the ALD treatment changes the surface wettability in an oxide- and cycle-dependent manner. In most cases, the surface becomes less hydrophilic compared to the R-cellulose, with higher contact angles observed for the samples prepared with 7 cycles. The higher contact angles at low cycle numbers are consistent with previous studies, where early stages of ALD growth have been associated with more hydrophobic surface behavior [17,30,31]. With increasing number of cycles, the surface properties shift again, which may be related to changes in surface composition and coverage.

The trend in contact angle does not directly follow the changes observed in the O–H absorption in FTIR. This difference can be explained by the different probing depths of the techniques. FTIR reflects contributions from both bulk and near-surface regions, whereas the contact angle is determined by the outermost surface. As a result, changes within the cellulose network may increase hydroxyl-related absorption without necessarily increasing the polarity of the outer surface.

### 3.2. Dielectric Properties

The dielectric properties of the samples were evaluated to assess how the ALD coatings influence the electrical response of the system (Table 1). The dielectric constant (ε′) varies depending on both the deposited oxide and the number of ALD cycles. While some variations are observed compared to the reference sample, no consistent trend across all materials and cycle numbers can be identified.

For the Al_2_O_3_ samples, the 7-cycle coating exhibits a slightly higher dielectric constant compared to the reference, while the 200-cycle sample shows a lower value. The TiO_2_- and ZnO-coated samples also show variations in dielectric constant compared to the reference, although no clear correlation with oxide type or number of ALD cycles was observed.

**Figure 7:**
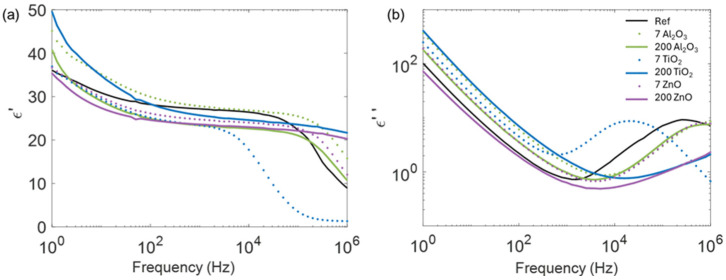
(**a**) Dielectric constant and (**b**) dielectric loss as a function of frequency for all samples.

The dielectric response also shows a clear frequency dependence (Figure 7). The dielectric constant (*ε′*) decreases with increasing frequency for all samples, which is typical for systems where polarization mechanisms cannot follow the applied field at higher frequencies. The differences between samples are more pronounced at low frequencies, while the values tend to converge at higher frequencies.

Although crystalline metal oxides often exhibit higher dielectric constants than their amorphous counterparts, the dielectric behavior in ALD-modified cellulose systems is substantially more complex and differs from conventional bulk dielectric composite systems where high-dielectric fillers are directly incorporated into the matrix. Since the oxide is deposited as an ultrathin conformal layer on a hygroscopic cellulose substrate, the effective dielectric response is influenced not only by oxide crystallinity, but also by interfacial polarization, oxide coverage, hydroxyl interactions, defect density, moisture interactions, possible precursor–cellulose interactions, and the dielectric properties of the underlying cellulose matrix. In heterogeneous systems such as these, charge accumulation at the oxide–cellulose interfaces may contribute to interfacial polarization effects, which can influence the overall dielectric response of the system.

The absence of a systematic trend therefore suggests that the dielectric response is governed by a combination of oxide-related and substrate-related effects rather than by the intrinsic dielectric properties of the deposited oxides alone. Since the ALD coatings constitute ultrathin surface modifications on a cellulose substrate, the measured dielectric constant likely reflects the effective dielectric response of the combined cellulose–oxide system rather than that of the deposited oxide itself.

The dielectric loss (*ε″*) similarly exhibits frequency-dependent behavior, with variations between the samples and indications of relaxation processes at intermediate frequencies. This may be related to interfacial and dipolar polarization within the system, although the exact contribution cannot be determined from the present data alone.

**Figure 8:**
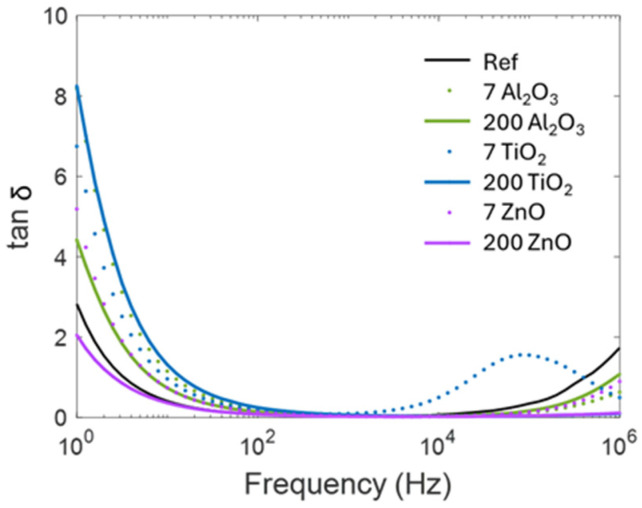
The dielectric loss tangent, tan *δ*, over different frequencies.

The dielectric loss tangent (tan *δ*) also shows clear differences between the samples at low frequencies (Figure 8). In the frequency range relevant for low-frequency operation (≈1–10 Hz), the R-cellulose and the 200-cycle ZnO sample exhibit relatively low tan *δ* values, indicating lower dielectric dissipation under these conditions. The TiO_2_-coated samples, on the other hand, show higher tan *δ* values at low frequencies, suggesting increased dielectric losses. The comparatively low dielectric loss observed for the 200-cycle ZnO sample may be related to the appearance of crystalline ZnO domains identified by XRD, although the exact relationship between crystallinity and dielectric behavior cannot be established from the present data alone.

In triboelectric systems, dielectric properties may influence charge generation, storage, and transfer behavior. A higher dielectric constant can contribute to enhanced charge storage capability, whereas increased dielectric loss may facilitate charge dissipation and reduce charge retention. However, in heterogeneous cellulose–oxide systems such as these, the relationship between dielectric behavior and triboelectric performance is likely governed by multiple interacting factors, including moisture interactions, defect states, interfacial effects, and charge trapping behavior at the oxide–cellulose interfaces. Similar relationships between dielectric behavior, charge trapping, and triboelectric performance have previously been reported in TENG systems [32].

### 3.3. Triboelectric Performance

**Figure 9:**
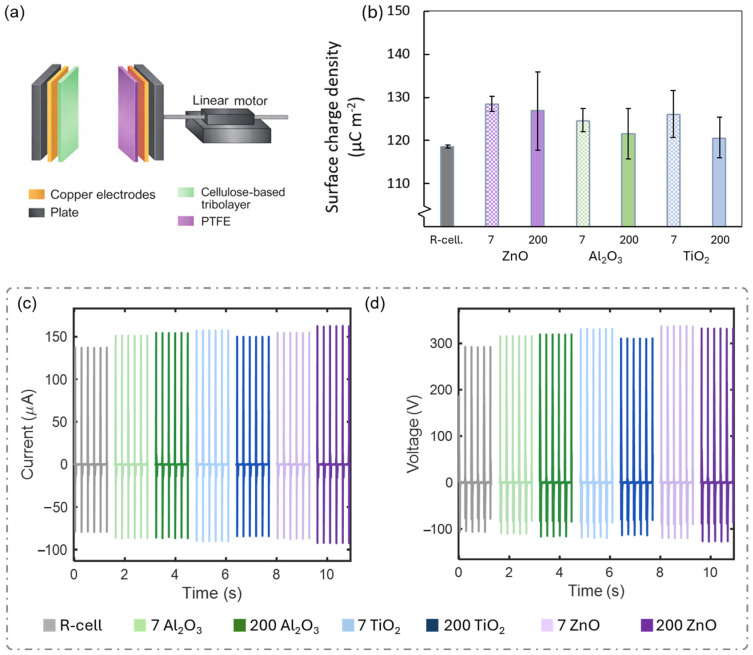
(**a**) Schematic illustration of the triboelectric nanogenerator (TENG) setup in the contact-separation mode. (**b**) Surface charge density for the regenerated cellulose film and the 7 and 200 cycle ALD of the three metal oxides. (**c**) The *I*_sc_ and (**d**) *V*_oc_ for all the samples.

The triboelectric performance of the ALD-modified cellulose films was evaluated using a contact–separation mode TENG configuration, where the cellulose-based tribolayer was paired with PTFE as the counter material (Figure 9a). The measurements were performed using a linear motor to enable comparison between the different ALD coatings and cycle numbers.

The surface charge density measurements (Figure 9b) show that all ALD-treated samples exhibit higher values compared to the untreated R-cellulose reference. The largest increase is observed for the 7-cycle ZnO sample, followed by the 200-cycle ZnO sample. For all investigated oxides, the samples prepared with 7 ALD cycles show slightly higher surface charge density compared to the corresponding 200-cycle samples.

Despite the relatively modest increase in surface charge density, all ALD-treated samples show improved triboelectric performance compared to the untreated R-cellulose. This indicates that regenerated cellulose is intrinsically triboelectrically active, while the ALD treatment mainly acts as a surface modification that affects the electrical output rather than fundamentally changing the charging behavior.

Figure 9c,d show representative short-circuit current (*I*_sc_) and open-circuit voltage (*V*_o*c*_) signals for the investigated samples.

**Figure 10:**
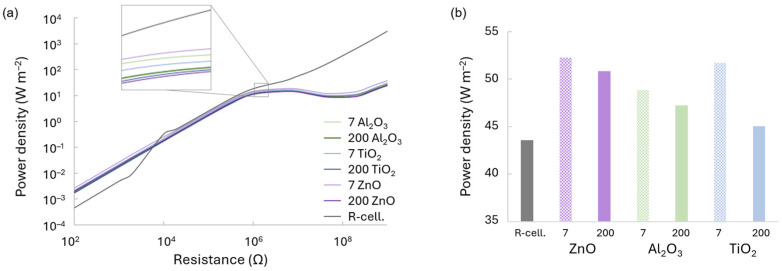
(**a**) Power output measured over different resistors and (**b**) the peak power output.

The R-cellulose film exhibits a distinctly different power–resistance characteristic compared to the ALD-modified samples, showing lower power density in the intermediate resistance range and a pronounced increase only at very high load resistances (Figure 10a). In contrast, the ALD-treated samples display a more defined impedance-matching region in the megaohm range, indicating differences in the electrical response of the triboelectric interface after ALD modification. These results suggest that ALD influences not only the surface charge density, but also the electrical characteristics governing power transfer in the triboelectric system [33].

The peak power output measurements (Figure 10b) closely follow the trends observed for surface charge density. All ALD-modified samples exhibit higher power output than the unmodified R-cellulose, with the highest values obtained for the 7-cycle ZnO coating. Increasing the number of ALD cycles to 200 results in lower power output for all investigated oxides, although the values remain above the R-cellulose reference level.

Differences among the ZnO, TiO_2_, and Al_2_O_3_ coatings may partially be related to their relative positions in the triboelectric series [34]. Although the exact triboelectric polarity depends on material pairing and surface conditions, ZnO consistently exhibited the highest power output, while TiO_2_ showed comparatively higher dielectric loss at low frequencies.

The ALD coatings also introduce oxide- and thickness-dependent differences in surface wettability (Table 1). However, no direct correlation between contact angle and peak power output is observed. For example, the 7-cycle ZnO sample exhibits higher performance than the more hydrophobic 200-cycle ZnO sample, indicating that wettability alone does not determine the triboelectric response. Likewise, neither dielectric constant nor dielectric loss alone fully explains the observed performance trends. Although oxide-dependent differences in dielectric behavior are observed, no single measured parameter shows a direct correlation with triboelectric output.

Instead, the results suggest that the early stages of ALD growth introduce near-surface and interfacial modifications that are sufficient to enhance the triboelectric response of regenerated cellulose. Increasing the number of cycles to 200 leads to a more developed oxide coating, but this does not further improve performance.

### 3.4. Humidity-Dependent Triboelectric Performance

**Figure 11:**
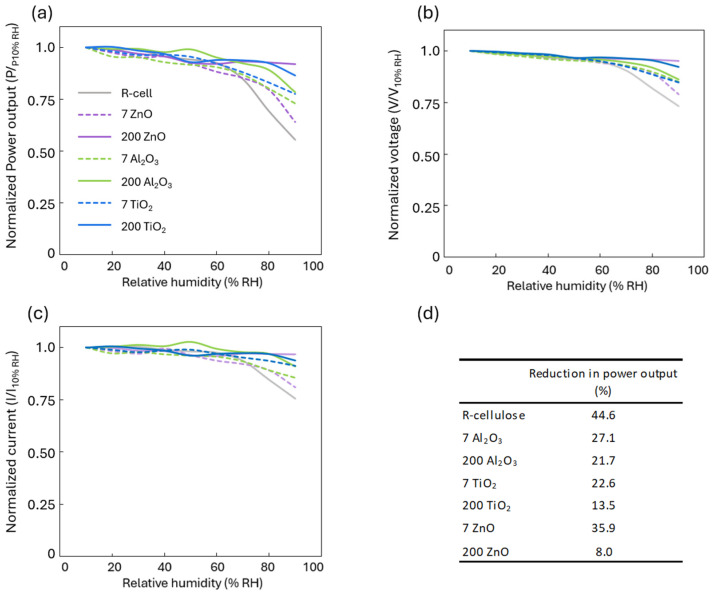
Humidity-dependent triboelectric performance of regenerated cellulose and ALD-modified samples measured between 10 and 90% RH. Normalized (**a**) peak power output $\left(P/P_{10\%RH}\right)$, (**b**) open-circuit voltage $\left(V/V_{10\%RH}\right)$, and (**c**) short-circuit current $\left(I/I_{10\%RH}\right)$ as a function of relative humidity. All values were normalized to the corresponding measurement obtained at 10% RH for each sample. (**d**) Relative reduction in peak power output at 90% RH compared to 10% RH.

To evaluate the influence of humidity on triboelectric performance, the power output of the samples was measured between 10 and 90% RH (Figure 11). The power output was normalized to the value measured at 10% RH for each sample to be able to follow the change.

All samples showed reduced output with increasing relative humidity, consistent with increased charge dissipation under humid conditions. However, the ALD-modified samples generally exhibited improved retention of triboelectric performance compared to the R-cellulose film at elevated humidity.

The largest decrease of 44.6% was observed for the R-cellulose sample, while the ALD-treated samples retained a larger fraction of their initial output. In particular, the ZnO sample coated with 200 cycles showed comparatively improved stability at high relative humidity and only decreased the power output with 8.0% at 90% RH.

**Figure 12:**
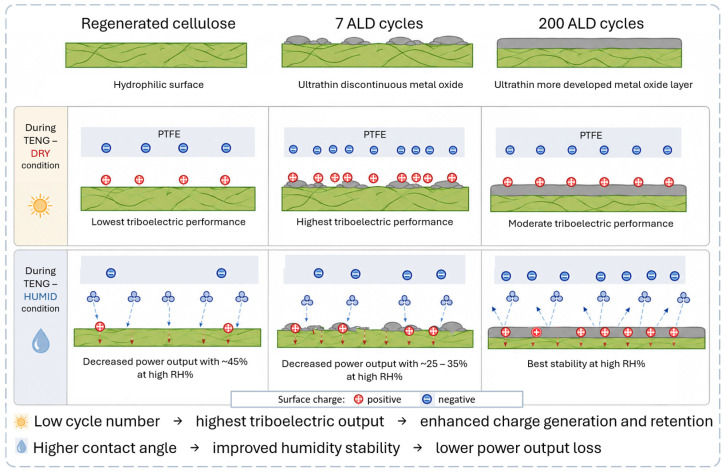
Proposed schematic illustration of how the ALD cycle number influences the triboelectric performance and humidity stability of regenerated cellulose-based TENGs. Low-cycle ALD coatings (7 cycles) resulted in the highest initial triboelectric output, while more developed oxide layers obtained after 200 cycles improved stability under humid conditions. The schematic is based on experimental observations and proposed interfacial effects and does not represent a proven mechanism.

Interestingly, the samples exhibiting the highest triboelectric output under low-humidity conditions were not necessarily those with the highest humidity stability. While the 7-cycle samples generally showed the highest initial performance, some of the more developed coatings exhibited improved output retention at elevated humidity. These results suggest that ALD modification influences not only the initial triboelectric response, but also the humidity-dependent electrical behavior of regenerated cellulose-based TENG systems. A schematic illustration of how the ALD cycle number influences the triboelectric performance and humidity stability of regenerated cellulose-based TENGs is shown in Figure 12.

The highest triboelectric output was observed for the 7-cycle ZnO sample, despite the absence of detectable ZnO crystallinity in XRD, whereas the 200-cycle ZnO sample exhibited improved stability at elevated humidity. This suggests that the triboelectric behavior is not governed solely by oxide crystallinity, but rather by a combination of interfacial modification, dielectric dissipation behavior, surface coverage, and humidity-dependent charge retention. The ultrathin ZnO modification introduced during the initial ALD growth stages may promote efficient charge generation without introducing excessive dielectric dissipation, while the more developed ZnO coating at 200 cycles may provide improved resistance to humidity-induced electrical losses through more continuous surface coverage and altered interfacial dielectric behavior. Similar relationships between dielectric engineering, interfacial charge trapping, and humidity-dependent triboelectric behavior have been discussed for oxide-containing and interfacial engineered TENG systems in previous studies [15,32].

## 4. Conclusions

This work systematically investigated the influence of ultrathin ALD metal oxide coatings on the triboelectric performance of regenerated cellulose films. By combining surface charge density measurements, power–load analysis, dielectric spectroscopy, wettability, and structural characterization, the results show that nanoscale interfacial modifications introduced by ALD influence the triboelectric response of regenerated cellulose.

All ALD-modified samples exhibited a modest but reproducible increase in surface charge density, further demonstrating that regenerated cellulose is intrinsically triboelectrically active and that ALD can serve as an effective surface modification strategy. Among the investigated oxides, ZnO exhibited the highest power output. The samples prepared with 7 ALD cycles consistently outperformed the corresponding 200-cycle samples.

Humidity-dependent measurements further demonstrated that the ALD coatings improved the retention of triboelectric performance at elevated relative humidity compared to pristine regenerated cellulose. While the uncoated R-cellulose sample exhibited the largest reduction in normalized power output, the ALD-modified samples retained a larger fraction of their initial performance. In particular, the 200-cycle ZnO sample showed the highest humidity stability.

Although oxide-dependent differences in dielectric behavior and wettability were observed, no single measured parameter showed a direct correlation with triboelectric performance. Instead, the results suggest that relatively small interfacial modifications introduced under the investigated low-cycle ALD conditions are sufficient to influence the triboelectric response and humidity-dependent charge dissipation behavior of regenerated cellulose-based TENG systems.

These findings provide insight into how ultrathin oxide coatings can be used to modify cellulose-based triboelectric materials and may support the development of more sustainable energy harvesting systems based on nanoscale surface engineering.

## Figures and Tables

**Table 1:** Thickness, dielectric constants (100 and 1000 Hz), contact angle, and surface roughness for all the samples.

	Sample Thickness (µm)	ε′ @10^2^ Hz	ε′ @10^3^ Hz	Contact Angle (°)	Surface Roughness R_q_ (nm)
R-cellulose	19.6 ± 0.5	28.2	27.2	54.5	14.7
7 Al_2_O_3_	27.2 ± 1.2	29.9	27.8	98.9	18.7
200 Al_2_O_3_	26.2 ± 1.2	24.9	23.4	81.5	10.1
7 TiO_2_	25.3 ± 1.0	25.1	23.4	91.6	11.6
200 TiO_2_	24.0 ± 1.3	28.3	25.7	84.4	14.3
7 ZnO	22.8 ± 0.8	26.1	24.7	71.3	11.3
200 ZnO	24.8 ± 0.8	24.6	23.6	106.9	15.2

## Data Availability

The data presented in this study are available on request from the corresponding author due to institutional data management practices.

## Abbreviations

The following abbreviations are used in this manuscript:

ALD	Atomic Layer Deposition
AFM	Atomic Force Microscopy
FTIR	Fourier-Transform Infrared Spectroscopy
PTFE	Polytetrafluoroethylene
SEM	Scanning Electron Microscopy
TENG	Triboelectric Nanogenerator
XRD	X-ray Diffraction
